# Using a Search Engine-Based Mutually Reinforcing Approach to Assess the Semantic Relatedness of Biomedical Terms

**DOI:** 10.1371/journal.pone.0077868

**Published:** 2013-11-13

**Authors:** Yi-Yu Hsu, Hung-Yu Chen, Hung-Yu Kao

**Affiliations:** 1 Department of Computer Science and Information Engineering, National Cheng Kung University, Tainan, Taiwan, Republic of China; 2 Institute of Medical Informatics, National Cheng Kung University, Tainan, Taiwan, Republic of China; University of Warwick, United Kingdom

## Abstract

**Background:**

Determining the semantic relatedness of two biomedical terms is an important task for many text-mining applications in the biomedical field. Previous studies, such as those using ontology-based and corpus-based approaches, measured semantic relatedness by using information from the structure of biomedical literature, but these methods are limited by the small size of training resources. To increase the size of training datasets, the outputs of search engines have been used extensively to analyze the lexical patterns of biomedical terms.

**Methodology/Principal Findings:**

In this work, we propose the Mutually Reinforcing Lexical Pattern Ranking (ReLPR) algorithm for learning and exploring the lexical patterns of synonym pairs in biomedical text. ReLPR employs lexical patterns and their pattern containers to assess the semantic relatedness of biomedical terms. By combining sentence structures and the linking activities between containers and lexical patterns, our algorithm can explore the correlation between two biomedical terms.

**Conclusions/Significance:**

The average correlation coefficient of the ReLPR algorithm was 0.82 for various datasets. The results of the ReLPR algorithm were significantly superior to those of previous methods.

## Introduction

Semantic relatedness has become increasingly important for the text-mining community in recent years, especially in the biomedical field. A large number of relationships between biomedical terms can now be produced at one time. For biomedical scientists, it is important to link the correct concepts to relevant diseases. For example, “renal failure” and “kidney failure” are distinct terms, but they refer to the same disease. However, there are various connections between different biomedical terms. Unlike the above example, “diarrhea” and “stomach cramps” are not identical and do not have similar relations, but “diarrhea” causes “stomach cramps”. Therefore, the existence of various relations has created additional challenges for understanding biomedical terms. Semantic relatedness is understood by human beings; however, this task is difficult for computers because most computational approaches require the efforts of human annotators to build the training dataset.

Most domain-specific resources are incomplete; therefore, several studies focus on domain-independent words [1], [2]. The quality and quantity of the corpus and ontology, particularly the shortage of domain-specific terms, affect the accuracy of evaluations. Many loanwords, such as “entropy”, are used in different domains, but these words can have various meanings. Therefore, the process of determining the semantic relatedness of domain-specific terms becomes difficult. The number of online databases of domain-specific terms has increased rapidly, and several studies have evaluated semantic relatedness in the biomedical sciences [3], [4]. Many terms that describe identical concepts are expressed as different words. For example, “Michigan Cancer Foundation - 7” can be abbreviated as “MCF-7”, and the two terms refer to the same cell line. In contrast, two terms might not refer to identical items, but they can be related to the same concept, such as “flu” and “bronchitis” or “estrogen” and “breast cancer”. Note that “flu” and “bronchitis” are both disorders of the respiratory system, and “estrogen” influences “breast cancer”.

Understanding the semantic relatedness of terms is a heuristic learning procedure, and the heuristic rules are based on the experience of annotators [5]. The standards used to assess biomedical terms are inconsistent because different annotators are specialists in different domains. For example, doctors believe that there is a strong relation between “congestive heart failure” and “pulmonary edema” because “pulmonary edema” leads to “congestive heart failure”. However, biologists consider the two terms to be different diseases and consider these terms to be unrelated. If a query term is excluded from ontology, its semantic relatedness cannot be evaluated [5].

In recent years, some studies have suggested that search engines can be used to study semantic relatedness [6], [7]. However, the research is still at an early stage of considering the co-occurrence and the similarity between two terms [8], [9]. Although semantic relatedness focuses on measuring the correlation between two terms, the connections between high and low semantic relations are left rather vague. Therefore, Pedersen et al. classified semantic relatedness into four levels [5]. These aforementioned definitions raised the possibility that a synonym pair provides the best description of a concept pair. Before evaluating the semantic relatedness of two terms, we retrieve their information from web resources; this information includes the co-occurrence of two terms, the distance of ontology, and the query results from search engines. Research studies on web resources focus on the following three types of applications for web sources: (a) ontology-based approach, (b) corpus-based approach, and (c) search engine-based approach.

### a. Ontology-based approach

The ontology can be represented as a hierarchical tree that describes the concepts and relations between vocabularies. In terms of ontology trees, a leaf node means a vocabulary, and an edge represents the relation between leaf nodes. Therefore, the ontology-based approach evaluates semantic relatedness by the distance between different nodes.

Rada et al. proposed an ontology-based approach for evaluating the semantic relatedness of biomedical literature [3]. The Medical Subject Headings (MeSH) ontology, developed by National Library of Medicine (NLM), has been used to define the relations of biomedical terms. For example, general concepts are the nodes that are close to the root. In contrast to general concepts, specific concepts are the nodes that are close to the leaves. The distance from a general concept to a specific concept is a measurement that indicates the relation between two terms. Moreover, the shortest paths on the ontology graphs have been examined using various forms of ontology-based approach [10]. Lord et al. used a gene ontology method [11] to evaluate the similarity of protein sequences [12]. The gene ontology is a logic system that classifies genes and proteins based on three classes: molecular function, biological process, and cellular component. The researchers assessed the similarity of two proteins by their annotated functions in the gene ontology, and the results were used to evaluate the sequence similarity of two proteins.

In considering the semantic relatedness of non domain-specific data, Wu et al. noted that two terms can share the same nodes from the root to the individual terms on their path in the ontology [13]. That is, semantic relatedness can be calculated from the similarity of the paths between two terms on ontology graphs. Furthermore, the semantic relatedness can be adjusted by the ratio of the shortest paths to twice the distance of the ontology [2].

Although the tree structures of ontology-based approaches can be used to measure semantic relatedness, they do not always deal with the following problem adequately. A problem occurs if a query term is missing from the ontology. In this case, there are no paths between a query term and a concept term; thus, it is impossible to determine the semantic relatedness. In contrast, the relations between terms can be simple, such as “is a”, but there is often more than one type of relation in the ontology. In fact, a single extra relation in the ontology increases the path length, limiting the ontology-based approach.

### b. Corpus-based approach

A corpus is a collection of large and structural contexts with many concepts. The corpus-based approach uses the contexts to obtain the relations of concepts and evaluates the semantic relatedness of concepts.

Resnik et al. assessed the semantic relatedness of concepts by the information content [14]. Information content measures the frequency with which a concept occurs in a large number of contexts. If the value of information content is high, the concept is considered as a specific one. In contrast to a specific concept, a general concept has a low information content value. To calculate the semantic relatedness of concept P and concept Q, Resnik et al. have employed WordNet [15] and the Brown corpus to compute the maximum value of information content for all concepts. However, many concepts share the same information content, causing the concepts to be similar. Therefore, several studies used the different information content of concepts to approximate the semantic relatedness [16], [17].

Wilbur and Yang used literature from PubMed and transformed the studies into a matrix; the matrix was then used to correlate the documents with their term frequency [18]. Moreover, the researchers evaluated the semantic relatedness of terms by the co-occurrence of the terms. Patwardhan and Pedersen employed a context vector to estimate the value of semantic relatedness [19], and they constructed the context vector from the literature by word sense discrimination [20] and latent semantic indexing [21]. Furthermore, the context vector is based on the co-occurrence of conceptual terms, and the cosine similarity is used to calculate the semantic relatedness of two conceptual terms. To estimate the semantic relatedness of a domain-specific corpus, Pedersen et al. measured the concepts by context vectors [5]. They used a biomedical corpus, “Mayo Clinic Corpus of Clinical Notes”, but the cost to build the indexing concepts was high, particularly for a large corpus.

### c. Search engine-based approach

The normalized Google distance (NGD) [8] uses the page counts in a search engine, and the NGD measures the scores of two distinct concepts. The NGD uses the page counts of each concept and the page counts of co-occurrence concepts. The NGD also includes the normalized information distance [22]. However, the NGD focuses on the co-occurrence of concepts on web pages, but the NGD does not consider the semantic relation between two concepts.

Sahami et al. illustrated how short text snippets of concepts in search engines affected semantic relatedness [6]. They assumed each snippet is a document and calculated its term frequency-inverse document frequency (TF-IDF). Then, the semantic relatedness of two terms was estimated by the inner product of the centroid vectors. Snippets can be used as part of a training strategy, but the snippets returned by search engines are often related to various concepts. The snippets produce noise because a concept is usually a polysemous word. Thus, the semantic relatedness changes dramatically with the collection of snippets.

Chen et al. used the double-checking model to evaluate the semantic relatedness between concepts [9]. The snippets of concept P and Q were collected, and both the frequency of the snippets of concept P in concept Q and the frequency of the snippets of concept Q in concept P were calculated. Although concept P and concept Q may be highly related according to the search engine ranking algorithms, the snippets of concept P do not provide empirical information about concept Q. In addition, the semantic relatedness of two concepts using Co-Occurrence Double Check is often zero.

Bollegala et al. used both page counts and short text snippets of search engines [7] and then evaluated the co-occurrence by four major measurements: the Jaccard coefficient, Overlap (Simpson), Dice, and Pointwise mutual information (PMI) [23]. At the same time, page counts were used to calculate the relation of concepts. The co-occurrence of two concepts does not correspond to the actual semantic relatedness. Therefore, Bollegala et al. proposed a framework for the semantic relatedness between concepts; this framework combines the lexical patterns from short text snippets with four characteristics of page counts. Then, the researchers used clusters of lexical patterns to improve the performance [1]. The semantic relatedness using search engine-based approaches for evaluating biomedical concepts has been discussed previously [4]. Chen et al. used four measurements of page counts and lexical patterns from known synonym datasets [24] to establish a rule-based system for comparing two concepts to measure the semantic relatedness.

**Figure 1:**
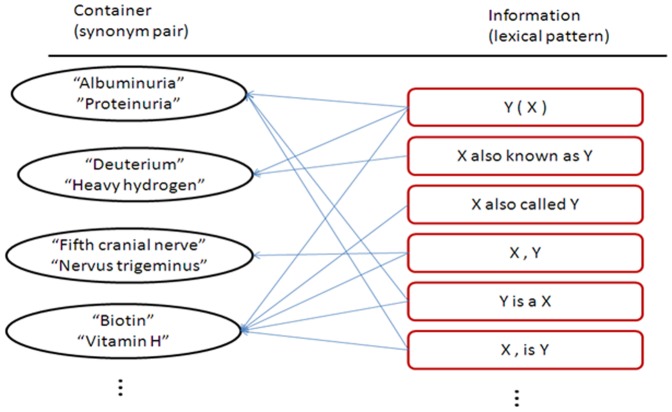
Synonym semantic relation networks.

The method proposed in this article employs known synonym pairs of biomedical data and the snippets from search engines. The semantic relation is termed information, whereas web pages, web sites, and a concept pair set are termed containers. Each container produces much information. Thus, synonym semantic relation networks are composed of the lexical patterns of the synonym relations of known concept pairs and containers, as shown in Figure 1. Using the idea of lexical semantic patterns, the linking analysis method evaluates concept pairs by comparing their lexical patterns with the lexical patterns of synonym semantic relation networks. To obtain the correlation scores of concept pairs, we calculate the overlap between the lexical semantic patterns of concept pairs and the lexical patterns of synonym semantic relation networks.

**Figure 2:**
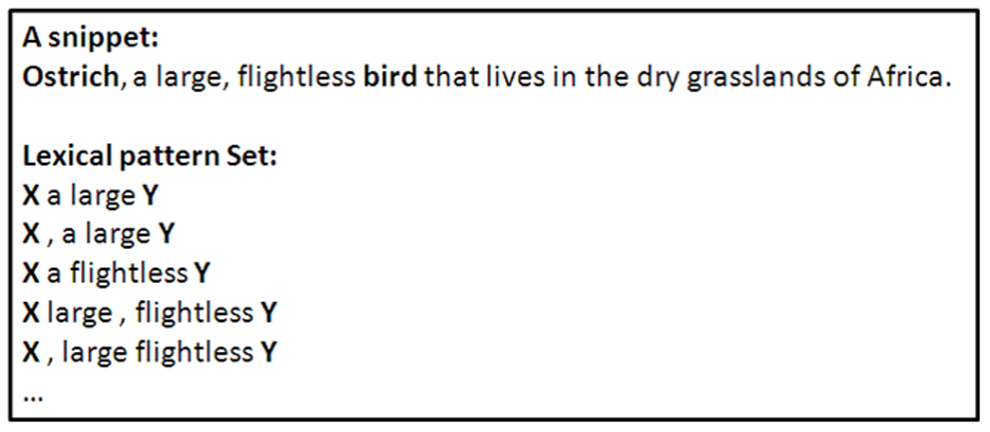
Lexical patterns of the concepts “ostrich” and “bird” from a search snippet.

Using search engines, we collected the web pages in which two terms “X” and “Y” both occur, and the resulting web page summaries are termed snippets. The snippets are queried with the syntax “X” + “Y”, and these snippets are the search results for phrases containing these two terms. We extracted the sentences that included the two terms and analyzed their structures, and then we examined the specific relations of the two terms. The extracted and analyzed forms are termed lexical patterns, and they indicate the semantic relation of the two terms. Figure 2 shows an example of building a lexical pattern set. If people attempt to understand the relation between “ostrich” and “bird”, they can query these terms in search engines and collect the snippets of the two terms. Then, they realize that “ostrich” is a member of “bird” by recognizing the lexical pattern, such as “X, a large Y”, and so on. If the specified relations of known term pairs have been constructed, we can evaluate unknown term pairs by retrieving their lexical patterns in search engines and comparing them with the lexical patterns of known term pairs. That is, an unknown term pair that has a larger number of specified lexical patterns has a higher possibility of the two terms being related. Consequently, we can evaluate the semantic relatedness of two terms by lexical patterns.

We define a set of lexical patterns within querying search engines as a container. A lexical pattern is a semantic relation of a term pair, and a semantic relation is comprised of different lexical patterns. Although the lexical patterns are under a specified semantic relation, the meanings of lexical patterns are distinct from each other. Here, we collected the lexical patterns with similar meanings as a container, and we divided the containers into three categories: page scope, website scope, and concept pair scope. Each scope contained the corresponding lexical patterns. The page scope refers to a container that collects the lexical patterns from web pages, and there are usually one or two lexical patterns in a web page. Moreover, the lexical patterns in a web page have similar forms because they are composed of the words and symbols in a single sentence.

**Figure 3:**
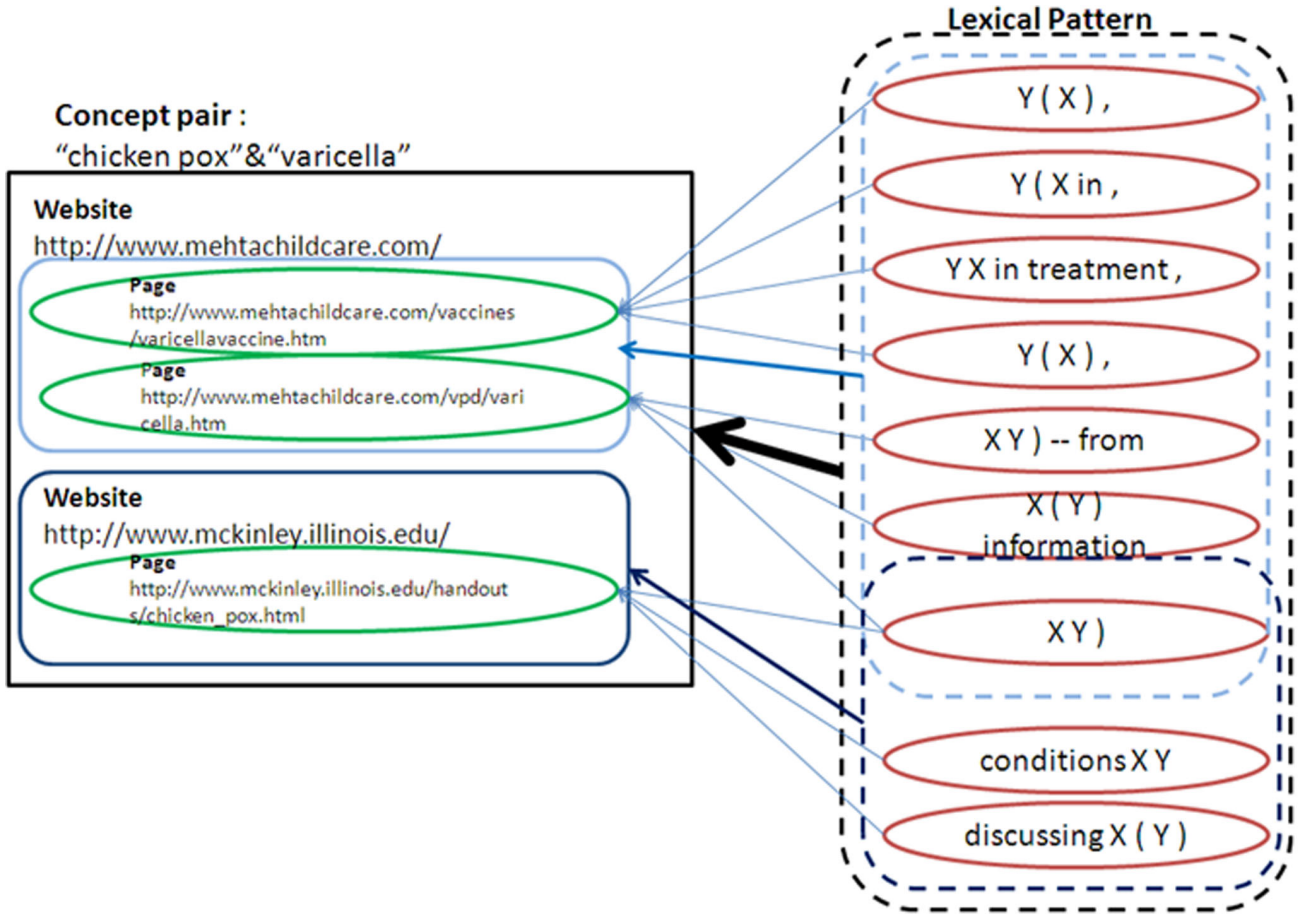
Container type scopes.

The lexical patterns of page containers are composed of repeated words and symbols, as shown in Figure 3. The website scope addresses the pages within a single website, and it constructs different lexical patterns from many pages. Hence, the more pages a website has, the more semantic information the website container has. Figure 3 shows the difference in the number of lexical patterns for website containers as the number of pages varies. As for the concept pair scope, the lexical patterns of a specified semantic relation can be collected from the pages of concept pairs. For a specified relation, the lexical patterns of a concept pair are dissimilar, and the number of lexical patterns for a concept pair reflects the variation in the semantic relations. For example, the lexical patterns of the concepts “chicken pox” and “varicella” are composed of the pages from different websites, as shown in Figure 3. The concept pair container is used to find meaningful lexical patterns for specified concept pairs.

**Figure 4:**
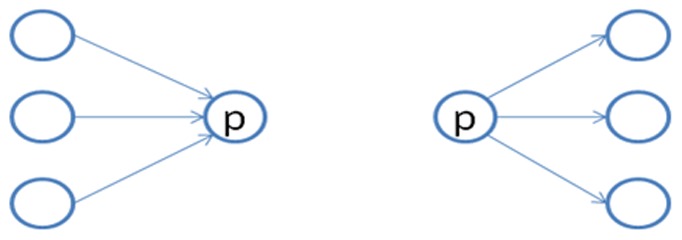
Authority and hub for web page p.

Here, we demonstrate the concept of the Hyperlink-Induced Topic Search (HITS) algorithm [25]. The HITS algorithm ranks the page by the linking structure of the networks. Each page has two values. One is the authority, known as the reputation of the web pages, and the other is the hub, known as the linking ability. The HITS algorithm develops an adjacency matrix to calculate the authority and hub recursively, as shown in Figure 4. Moreover, the authority of web page P is the summation of all the hubs of web page P, and the hub of web page P is the summation of all the authorities of web page P.

**Figure 5:**
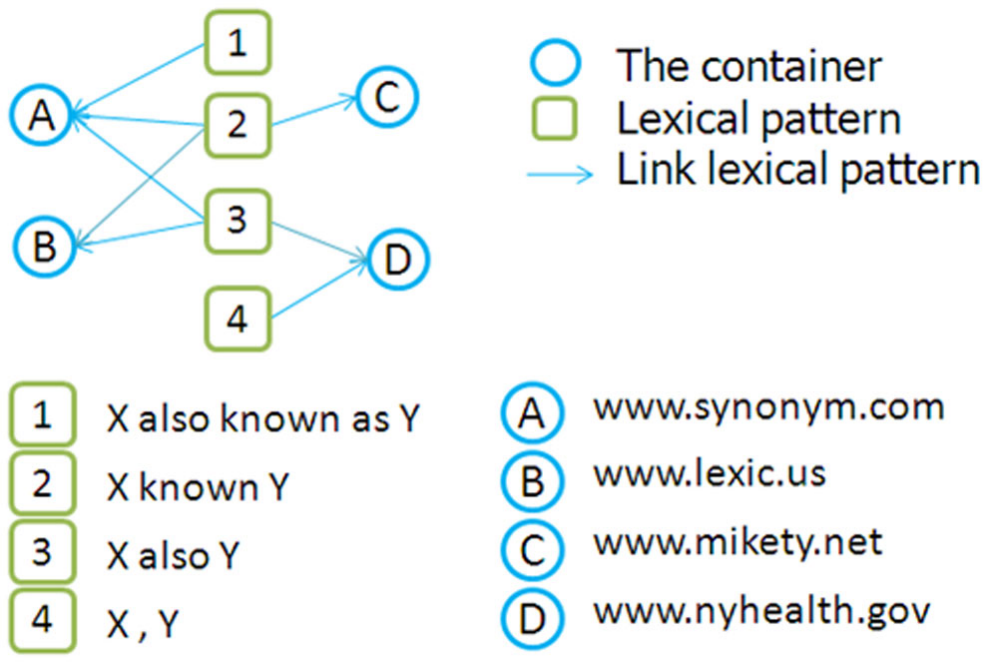
Lexical patterns - Container networks.

The container is regarded as a specified relation, and the information in the container is considered a specified feature. Each feature is capable of distinguishing different relations; thus, the container information combines the specified relations with networks. Thus, we apply the HITS algorithm to search the networks of containers, and then we propose a lexical pattern-voting method to identify the important container, as shown in Figure 5. In the lexical pattern-voting method, the importance of a given container is identified with its lexical pattern. In Figure 5, the network is composed of four lexical patterns and four containers. After applying the HITS algorithm to the network, we obtain the following rank: lexical pattern of 3 > lexical pattern of 2 = lexical pattern of 1 > lexical pattern of 4. The results show that the lexical pattern of 3 has the best performance for finding the relations between concepts while the lexical pattern of 4 has the worst performance. After applying the HITS algorithm to the networks of snippets, we can rank the lexical patterns as follows: lexical pattern 3 > lexical pattern 2 = lexical pattern 1 > lexical pattern 4.

## Materials and Methods

In this work, we propose a method to measure the semantic relatedness between two biomedical terms. The snippets of biomedical synonym pairs are collected from search engines, and the lexical patterns are constructed from the snippets. Here, we apply the lexical-pattern-voting method to identify the strong lexical patterns from the generated lexical pattern sets. The Mutually Reinforcing Lexical Pattern Ranking (ReLPR) algorithm is designed for learning the lexical patterns of biomedical synonym pairs, and it determines the semantic relatedness of concept pairs.

**Figure 6:**
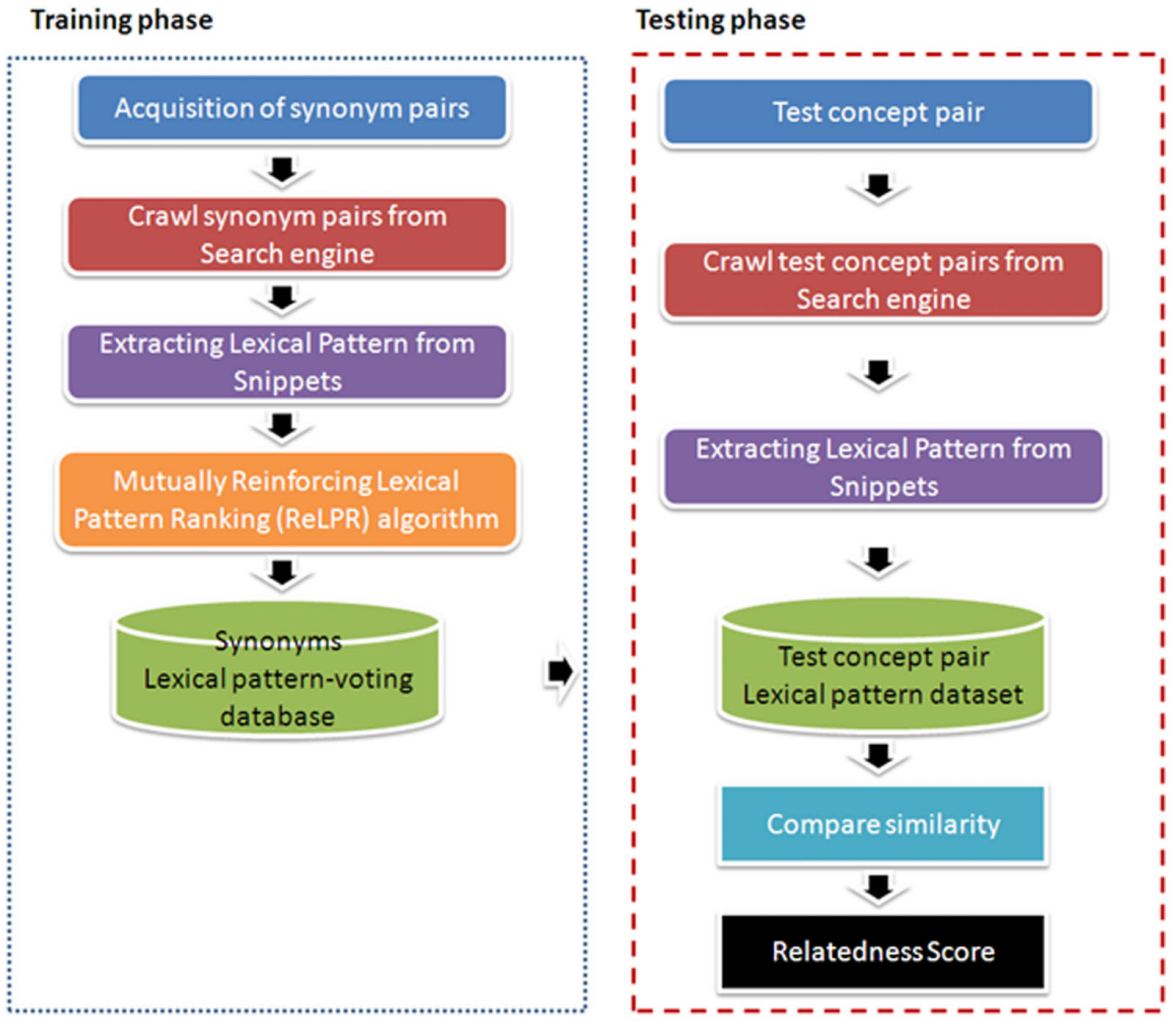
System flowchart.

The framework of this work is shown in Figure 6. There are four stages of training a synonym lexical pattern database: (1) the synonym pairs are collected; (2) the snippets of synonym pairs are retrieved from search engines; (3) the scores of lexical pattern-voting are extracted by the ReLPR algorithm for each lexical pattern; and (4) a synonym lexical pattern database is constructed. When the lexical pattern database is ready, the semantic relatedness of concept pairs can be evaluated from their lexical patterns. As mentioned above, we can extract the patterns of concept pairs and compare them with the synonym lexical patterns in the database. The overlap of lexical patterns between concept pairs and synonym pairs is related to the semantic relatedness. The method is structured as follows. First, we describe how to access the training data. Next, we demonstrate how to retrieve snippets from search engines. Then, the process of constructing lexical patterns is presented, and we apply the ReLPR algorithm to generate lexical pattern-voting candidates with the snippets of synonym pairs. Finally, we propose a ranking algorithm to compare a pair of biomedical terms.

### Acquisition of synonym pairs

To determine the semantic relatedness between concepts, a concept is defined as the synonym pair with the highest correlation [24], and the semantic relations in a synonym pair have specified forms. For example, concept P and concept Q have the following forms: “P(Q)”, “and P(Q”, “,P(Q”, “P known as Q”, “P/Q”, and “P,Q”. However, there are many semantic relations of a synonym pair, and the semantic relations are distinct for different synonym pairs. Thus, a concept pair can be considered to be a highly related pair when the concept pair has a large number of the semantic relations that are found for synonym pairs. In this case, synonym pairs of known biomedical terms are prepared to determine the semantic relations of synonyms.

The known biomedical terms were collected from MedicineNet.com, and the synonyms were collected from Synonyms.net. Both websites provide users with on-line dictionaries and query systems. Therefore, we designed an automatic retrieval program to integrate the resources from both websites, and the results are considered the known synonym pairs.

### Crawl concept pair from a search engine

**Figure 7:**
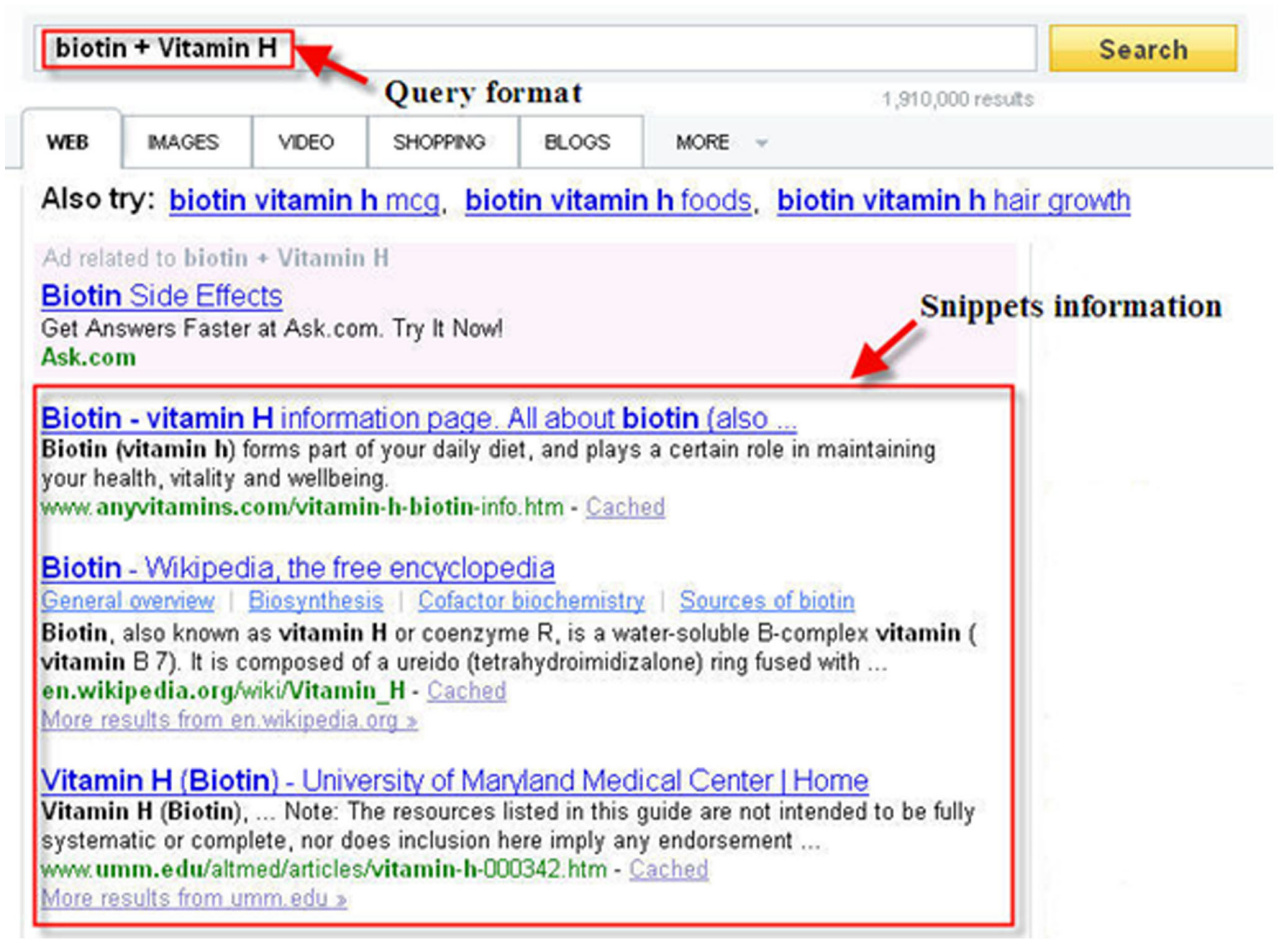
Query format “biotin”+ “vitamin H” in search engines.

Biomedical information has been increasing sharply over the past few years. For querying enormous amounts of data, search engines are important for retrieving the corresponding data in a short time. When users query keywords in search engines, search engines return the page counts and the contexts with keywords (snippets). Furthermore, the snippets are very useful because users can access the snippets without downloading the data from the original websites. Thus, users can effectively obtain the necessary information by scanning the snippets. To utilize these advantages of search engines, we queried two concepts and collected their snippets from search engines. However, a concept usually consists of many words, and the snippets are limited by the query format of the search engine. To obtain snippets that included two concepts, we used a union query to collect the snippets in which both concepts occur together. For example, we queried “biotin”+ “vitamin H” and collected the snippets, as shown in Figure 7. After retrieving the snippets, we can investigate the structure of the patterns.

### Extracting Lexical Pattern from Snippets

**Figure 8:**
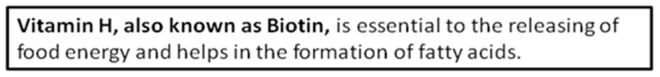
A snippet retrieved from the concept pair “biotin” and “vitamin h”.

After retrieving snippets, we must determine the semantic relations between two concepts. As shown in Figure 8, there are two concepts in a snippet, and “, also known as” is a semantic relation between biotin and vitamin H. If two concepts are synonymous, there are specific semantic relations between them. To find the semantic relations of snippet sets, we constructed the lexical semantic pattern to represent the semantic relatedness of two concepts. A previous study [7] indicated that semantic relations are often identified using the following process. First, the snippets of concept A and concept B are collected, and then concept A and concept B are used instead of variables X and Y. Next, the sequence set is generated by an n-gram sliding window, and the terms inside of the punctuation marks are considered to be a word. Then, the sequence is selected using the following rules.

The sequence includes one X and one Y.
The longest sequence length is L words, and the shortest sequence length is l words.
The sequence is allowed to skip g consecutive words. The skipped words of the sequence are the G words.

Finally, we consider the sequences of snippets to be lexical semantic pattern sets, as shown in Figure 8. For example, the lexical semantic pattern sets of two concepts are distilled, such as “Y also known as X”, “Y, known as X”, “Y, also as X”, “Y, also known X”, “Y known as X”, “Y also as X”, “Y also known X”, and “Y known as X is”.

### ReLPR: Mutually Reinforcing Lexical Pattern Ranking algorithm

Each lexical pattern in a synonym pair represents a characteristic of synonymous relations, but it is difficult to assess a synonym pair using only one lexical pattern. For example, the lexical pattern “X also called Y” between concept X and concept Y () is a synonymous relation. In contrast, the lexical pattern “X of Y” is ambiguous about whether the relation is synonymous. Computers are incapable of recognizing which lexical patterns distinguish synonym pairs. Hence, we used the lexical patterns that were generated from known synonym pairs to learn the lexical patterns of synonymous relations.

**Figure 9:**
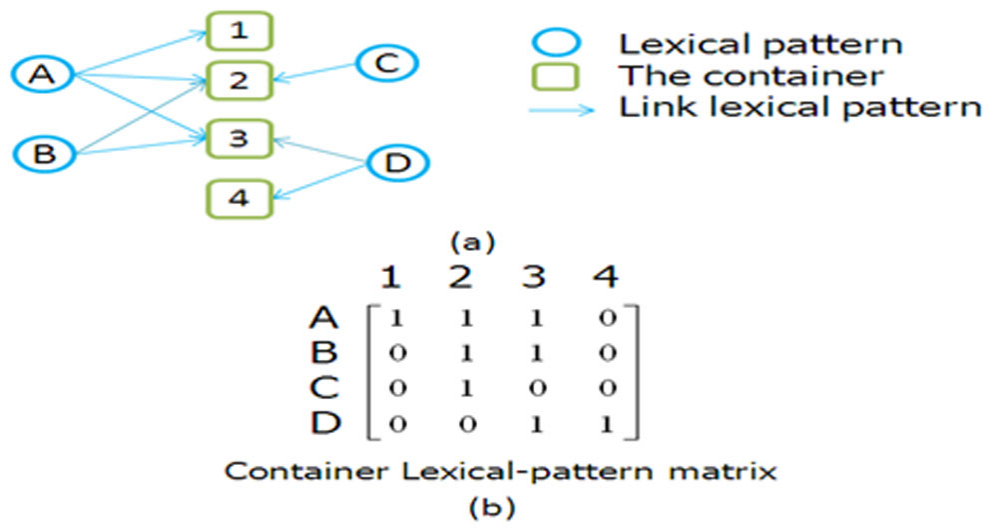
Lexical pattern (a) - container network (b) Container Lexical - pattern matrix.

A snippet is derived from a page, and many pages constitute a website. When searching for a concept pair in search engines, we obtain a collection of web pages. Thus, web pages, websites, and concept pairs can be regarded as a container with many lexical patterns. For example, a snippet page of “biotin” and “vitamin H” can produce the following patterns: “Y also known as X”, “Y, known as X”, “Y, also as X”, “Y, also known X”, “Y known as X”, as shown in Figure 8. Note that networks are composed of containers and lexical patterns, which include two matrices, the container matrix and the lexical pattern matrix, as shown in Figure 9. In Figure 9, (a) indicates a network that consists of four containers and four lexical patterns, and (b) is for a Container Lexical-pattern matrix, which shows that a lexical pattern in a container is given a weight if it has an in-link; otherwise, the lexical pattern is assigned a weight of zero. In a transposed Container Lexical-pattern matrix, a lexical pattern in a container is given a weight if it has an out-link; otherwise, the lexical pattern is assigned a weight of zero. In addition, the weights of the in-link and the out-link are adjusted by the number of lexical patterns and containers.

**Figure 10:**
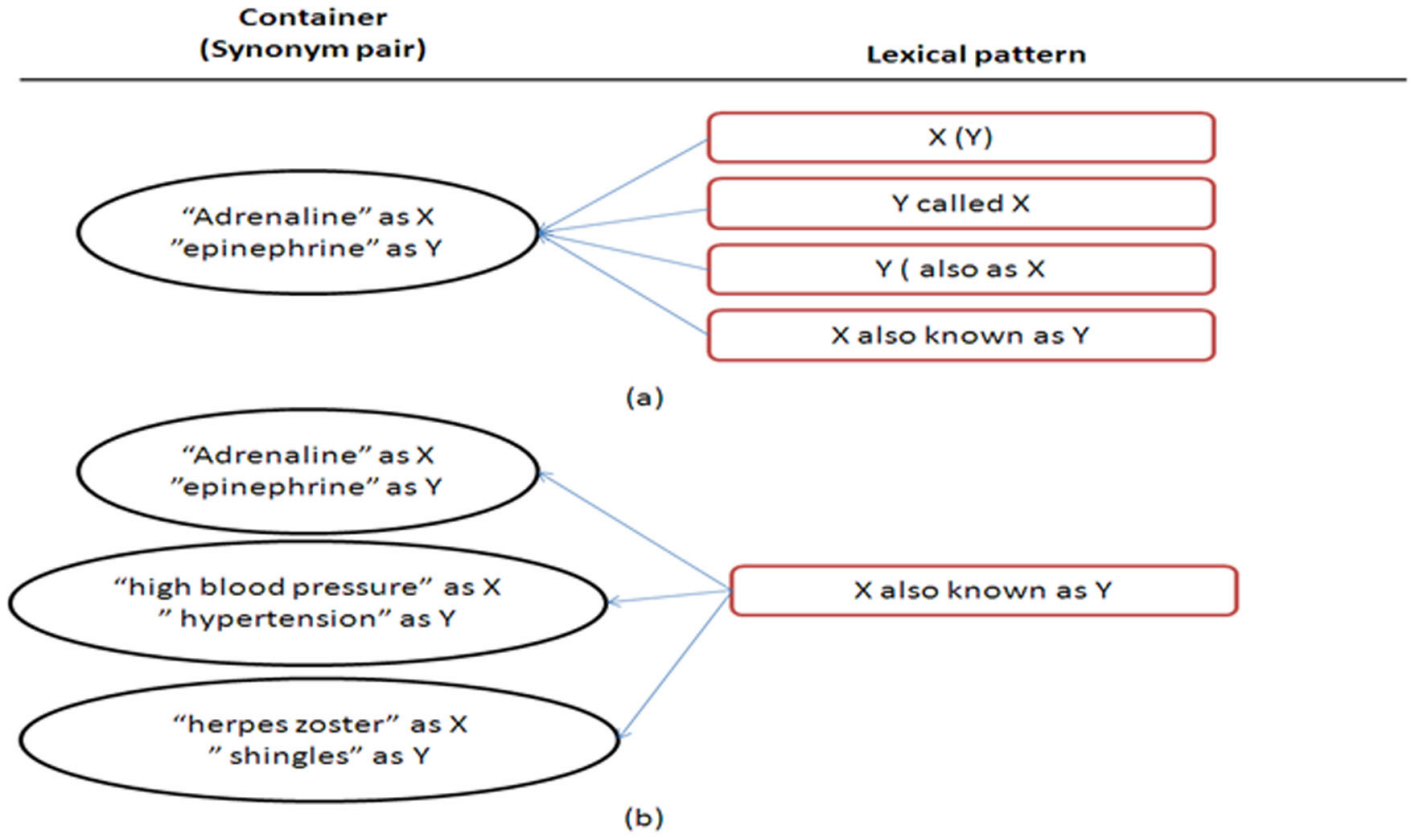
A container links to lexical patterns(a). A lexical pattern is connected by containers (b).

The lexical patterns are determined by their containers; thus, the networks of containers play important roles when analyzing the strengths of lexical patterns. Under the link analysis view, the ReLPR algorithm is similar to the HITS algorithm, and the lexical pattern-voting candidates are generated by the linking structure of the containers. The importance of a container is determined by its number of lexical patterns. In Figure 10 (a), the lexical patterns of “adrenaline” (X) and “epinephrine” (Y) include the following: “X (Y)”, “X also known as Y”, “Y (also as X)”, and “Y called X”. The strengths of lexical patterns are based on the number of occurrences within containers, as shown in Figure 10 (b). For example, “X also known as Y ” occurs in “adrenaline and epinephrine”, “high blood pressure and hypertension”, and “herpes zoster and shingles”.

**Figure 11:**
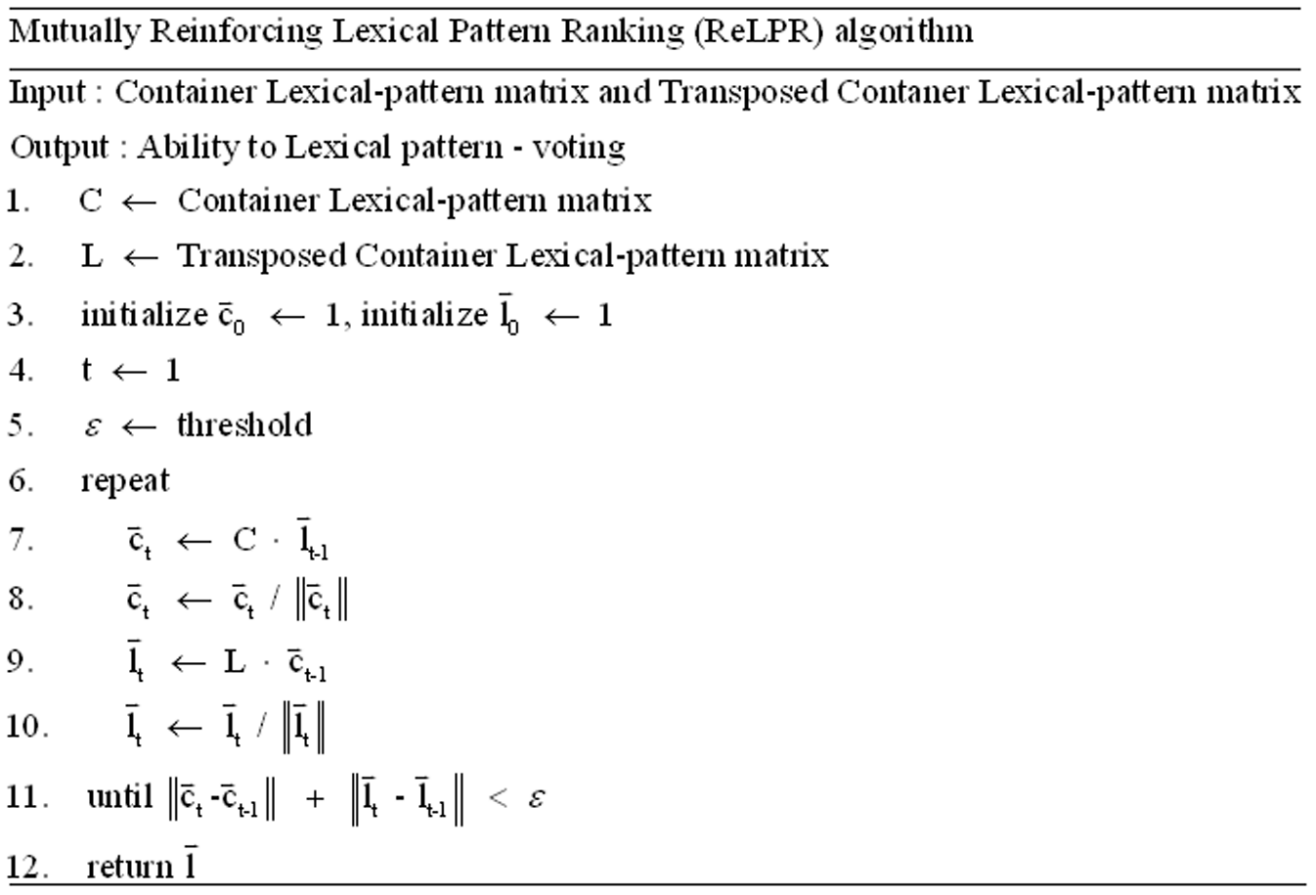
Mutually Reinforcing Lexical Pattern Ranking algorithm.

The ReLPR algorithm is shown in Figure 11. ReLPR increases the number of lexical patterns by a strategy of learning known synonym datasets, and a reinforcing method is applied to estimate semantic relatedness based on the influence between lexical patterns and pages. First, the container matrix and the lexical pattern matrix are initialized to 1, and they are matched with each other. Next, the container matrix and the lexical pattern matrix are iteratively modified by their influence on the partner matrix. For the scores that are calculated using the lexical pattern-voting approach, the algorithm is convergent when the difference between the container matrix and the lexical pattern matrix is smaller than a threshold ε. That is, stronger synonymous relations co-occur in the more important containers, and these lexical patterns are capable of distinguishing synonymous relations.

### Measuring Semantic Relatedness

After evaluating the semantic relatedness of a concept pair, we used the lexical patterns of known concept pairs to estimate their similarity. Therefore, we also queried the concept pairs in search engines, and the snippets of concept pairs were collected. After retrieving the snippets, we constructed the lexical patterns from the snippets, and these patterns were the semantic forms of concept pairs. However, these patterns were unable to determine the semantic relatedness of concept pairs. Here, we used the synonym lexical pattern database to compare the lexical patterns of unknown concept pairs with those of known concept pairs, and we calculated their similarity by the following equation:
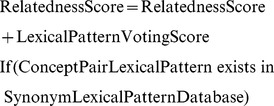
(1)


If the lexical patterns of unknown concept pairs matched the lexical patterns in the synonym lexical pattern database, we assigned a lexical pattern-voting score to judge the correlation between unknown and known concepts in terms of the semantic relations of synonym pairs. Hence, the semantic relatedness of a concept pair was obtained after we combined the lexical pattern-voting score with the calculations of concept pairs. That is, the more features of synonym pairs that a concept pair has, the higher the possibility that a concept pair is related to a synonym pair.

## Results and Discussion

### Dataset

Estimating the semantic relatedness of biomedical terms is difficult for untrained individuals. Therefore, the task of recognizing biomedical terms should be assigned to domain-specific experts. As far as we know, manual benchmark datasets are rare. Here, dataset 1 [26] includes 36 concept pairs of biomedical terms, and dataset 1 was marked by a group of doctors. In contrast, dataset 2 [5] consists of 30 concepts pairs of biomedical terms, and dataset 2 was marked by three doctors and nine biomedical experts, as shown in Table 1 and Table 2. In addition, we produced 1482 biomedical synonym pairs as our training sets (Supporting Information at http://ikmbio.csie.ncku.edu.tw/ReLPR/Supporting_Information/).

**Table 1:** Biomedical concept pairs of Dataset 1.

Term A	Term B	Doctor Score
Chicken Pox	Varicella	0.968
Antibiotics	Antibacterial Agents	0.937
Measles	Rubeola	0.906
Malnutrition	Nutritional Deficiency	0.875
Down Syndrome	Trisomy 21	0.875
Pain	Ache	0.875
Seizures	Convulsions	0.843
Breast Feeding	Lactation	0.843
Myocardial Ischemia	Myocardial Infarction	0.750
Carcinoma	Neoplasm	0.750
Migraine	Headache	0.718
Urinary Tract Infection	Pyelonephritis	0.656
Failure to Thrive	Malnutrition	0.625
Psychology	Cognitive Science	0.593
Vaccines	Immunity	0.593
Hepatitis B	Hepatitis C	0.562
Pulmonary Valve Stenosis	Aortic Valve Stenosis	0.531
Diabetic Nephropathy	Diabetes Mellitus	0.500
Hypertension	Kidney Failure	0.500
Lactose Intolerance	Irritable Bowel Syndrome	0.468
Sickle Cell Anemia	Iron Deficiency Anemia	0.437
Adenovirus	Rotavirus	0.437
Hypothyroidism	Hyperthyroidism	0.406
Sarcoidosis	Tuberculosis	0.406
Asthma	Pneumonia	0.375
Neonatal Jaundice	Sepsis	0.187
Hyperlipidemia	Hyperkalemia	0.156
Osteoporosis	Patent Ductus Arteriosus	0.156
Bacterial Pneumonia	Malaria	0.156
Otitis Media	Infantile Colic	0.156
Amino Acid Sequence	Antibacterial Agents	0.156
Acquired Immunodeficiency Syndrome	Congenital Heart Defects	0.062
Dementia	Atopic Dermatitis	0.062
Anemia	Appendicitis	0.031
Meningitis	Tricuspid Atresia	0.031
Sinusitis	Mental Retardation	0.031

**Table 2:** Biomedical concept pairs of Dataset 2.

Term A	Term B	Physician	Expert
Renal failure	Kidney failure	4	4
Heart	Myocardium	3.3	3
Stroke	Infarct	3	2.8
Abortion	Miscarriage	3	3.3
Delusion	Schizophrenia	3	2.2
Congestive heart failure	Pulmonary edema	3	1.4
Metastasis	Adenocarcinoma	2.7	1.8
Calcification	Stenosis	2.7	2
Diarrhea	Stomach cramps	2.3	1.3
Mitral stenosis	Atrial fibrillation	2.3	1.3
Chronic obstructive pulmonary disease	Lung infiltrates	2.3	1.9
Rheumatoid arthritis	Lupus	2	1.1
Brain tumor	Intracranial hemorrhage	2	1.3
Carpel tunnel syndrome	Osteoarthritis	2	1.1
Diabetes mellitus	Hypertension	2	1
Acne	Syringe	2	1
Antibiotic	Allergy	1.7	1.2
Cortisone	Total knee replacement	1.7	1
Pulmonary embolus	Myocardial infarction	1.7	1.2
Pulmonary fibrosis	Lung cancer	1.7	1.4
Cholangiocarcinoma	Colonoscopy	1.3	1
Lymphoid hyperplasia	Laryngeal cancer	1.3	1
Multiple sclerosis	Psychosis	1	1
Appendicitis	Osteoporosis	1	1
Rectal polyp	Aorta	1	1
Xerostomia	Alcoholic cirrhosis	1	1
Peptic ulcer disease	Myopia	1	1
Depression	Cellulites	1	1
Varicose vein	Entire knee meniscus	1	1
Hyperlipidemia	Metastasis	1	1

### Evaluation criteria

The evaluations of the semantic relatedness between terms can be divided into direct and indirect assessments. The direct assessment measures the difference between systematic methods and manual benchmarks. In contrast to direct assessments, indirect assessments apply the results to the article classifiers and recommender systems, and the performance is often evaluated according to the systematic view. As a result, we used direct assessments to evaluate the performance by two benchmark datasets. Here, the Pearson's correlation coefficient between the results and two benchmark datasets is given as follows.
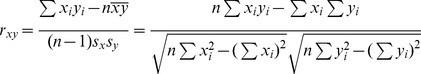
(2)where 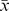 is the sample mean, and 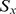 is the sample standard deviation.

### Analysis of training set

**Figure 12:**
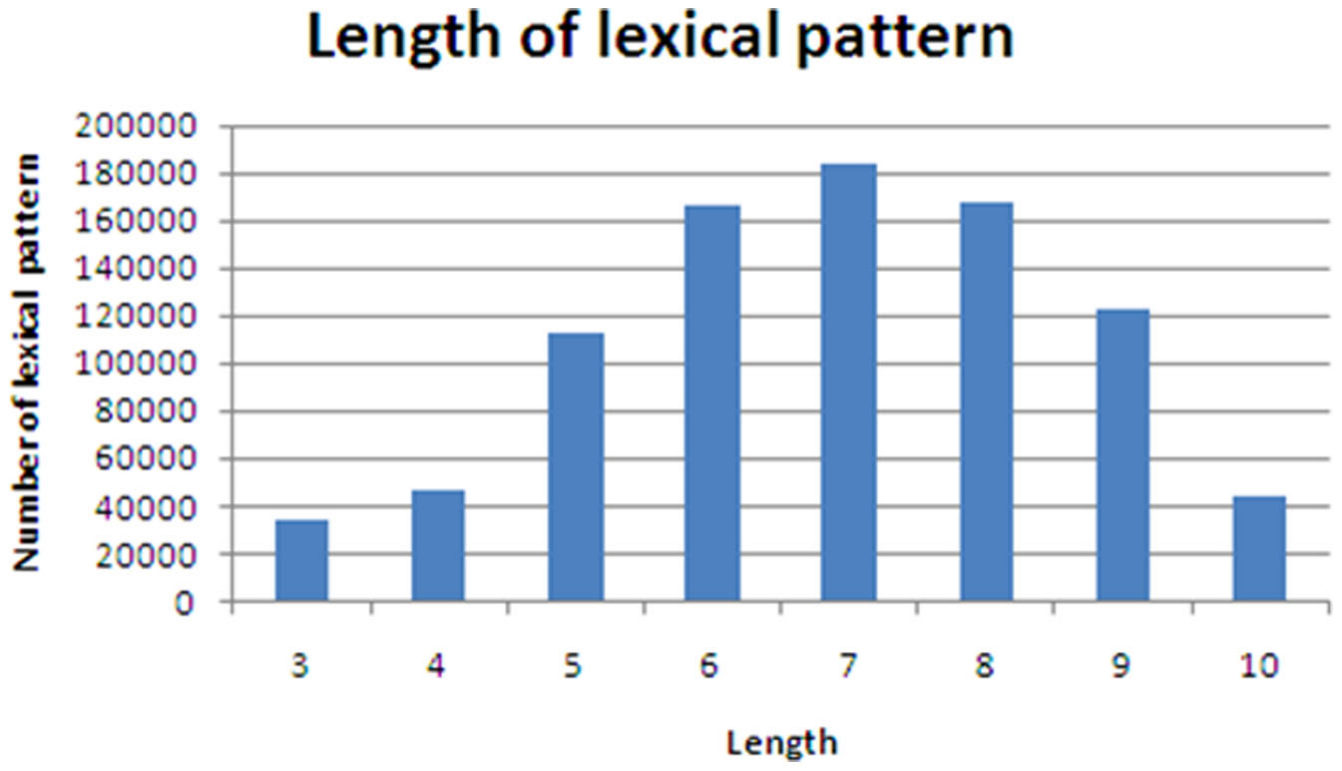
Distribution of lexical pattern length.

**Figure 13:**
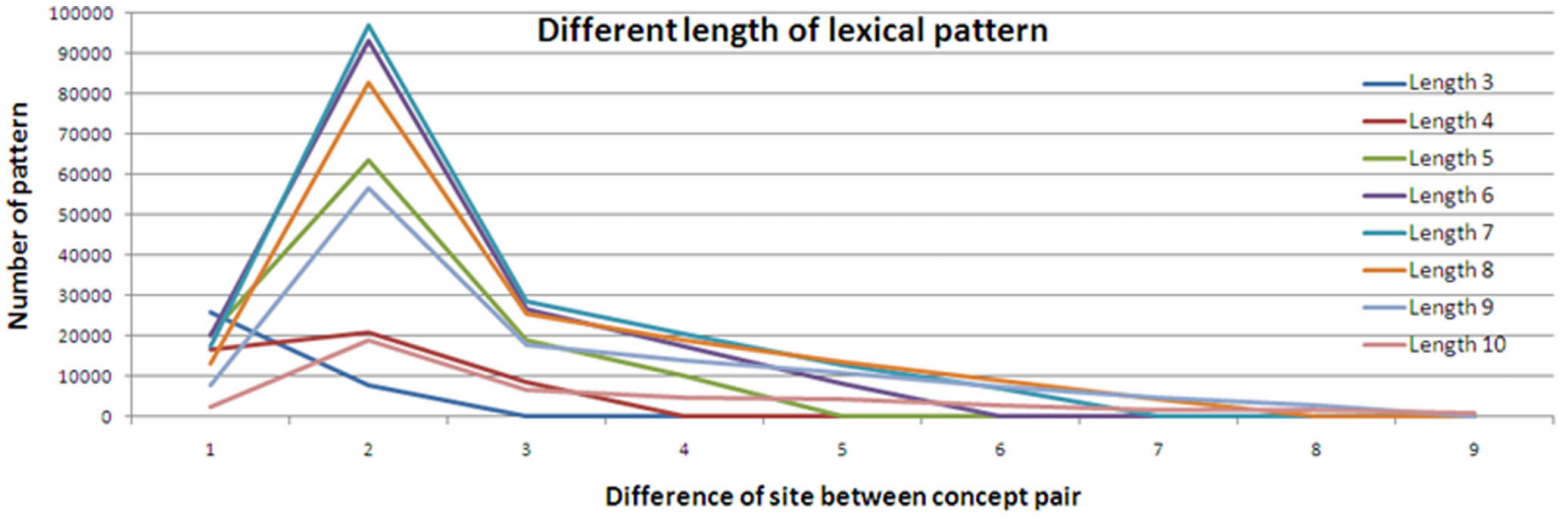
Number of different sites between concept pairs in lexical patterns.

The length of a lexical pattern is important for deciding the number of lexical patterns. To observe the influence of the lexical pattern length, we utilized several length parameters to assess the distributions of lexical patterns, as shown in Figure 12. However, the lexical pattern length was only reflected in the number of lexical patterns. Hence, we analyzed the distance between two terms, and we found that there are usually only three of fewer symbols or words between two terms, as shown in Figure 13. Furthermore, we observed the top 5 lexical patterns with different lengths, as shown in Table 3. Shorter lengths correspond to more concentrated lexical patterns. Although there are many long lexical patterns, most of the lexical patterns in the TOP 5 are short. That is, the lexical patterns with long lengths have changed dramatically. Furthermore, we also noticed that the longer lexical patterns are comprised of shorter lexical patterns. For example, “X, also known as Y, is” is from “X, also known as Y”. Therefore, we set the length parameter to L = 5 to recognize the words between two terms and distilled the lexical patterns.

**Table 3:** Top 5 Lexical patterns of different lengths.

Length	Top 5 lexical pattern	Frequency
3	X , Y	1324
	Y , X	1228
	X Y ,	1038
	X (Y	872
	Y X ,	811
4	X , Y ,	1092
	, X , Y	885
	Y , X ,	867
	X (Y)	692
	, Y , X	678
5	, Y , X	678
	, X , Y ,	647
	, Y , X ,	474
	X (Y) ,	275
	X , Y – (	254
6	Y , or X ,	246
	(noun) X , Y	234
	X , or Y , is	164
	X, also known as Y	147
	Y , or X , is	143
7	X (Y) is a	140
	X, also known as Y ,	123
	s: (n) X , Y	118
	what does X mean ?) Y	116
	Y , also known as X ,	95
8	(what does X mean ?) Y	116
	X, also known as Y , is	98
	(n) X , Y (	94
	s: (n) X , Y (	87
	(what does Y mean ?) X	82
9	Y , also known as X , is	73
	X , also known as Y , is a	63
	Y , also known as X , is a	43
	Y - also known as or related to X	36
10	s: (n) X , Y (a	25
	Y- also known as or related to X ,	14
	choose quality X Y manufacturers , suppliers , exporters at	13
	X - also known as or related to Y ,	13
	X (generic name - Y) online information -	10
	the X , also known as the Y , is	10

We then observed the differences in the lexical patterns and filtered out words between terms to measure the diversity of lexical patterns. When distinct words and symbols are removed, the lexical patterns become similar. For example, “Y, also known as X” can be generated from “Y, also known as or related to X” and “Y, also known as X,” by displacing the words. Hence, we can obtain the specified lexical patterns of synonym pairs by filtering out various words and symbols. Table 4 lists the different parameter settings to generate specified lexical patterns: (a) filter out one word or symbol from lexical patterns, (b) filter out two words or symbols from lexical patters, and (c) filter out three words or symbols from lexical patterns. Using the above parameter settings, we created the distributions of the Top 5 lexical patterns. For all parameter settings, we obtained similar lexical patterns in the Top 5. In (c), we observed that there are several common specified lexical patterns. For example, “X also known as Y” is a known lexical pattern for a synonym pair. Therefore, we set the filtering parameters G and g to be G = 3 and g = 2.

**Table 4:** Top 5 Difference between skipping parameter sets.

Length	Top 5 Lexical pattern	Frequency	Length	Top 5 Lexical pattern	Frequency	Length	Top 5 Lexical pattern	Frequency
3	X , Y	1212	3	X , Y	1286	3	X , Y	1231
	Y , X	1016		Y , X	1187		Y , X	1066
	X (Y	700		X Y ,	1087		X Y ,	909
	Y (X	604		Y X ,	885		Y X ,	691
	X ; Y	601		X (Y	833		X (Y	679
4	X , Y ,	866	4	X , Y ,	1043	4	X , Y ,	856
	Y , X ,	599		Y , X ,	865		Y , X ,	666
	X (Y)	590		X (Y)	706		X (Y)	573
	Y (X)	515		, X , Y	682		, X , Y	543
	, X , Y	447		Y (X)	646		Y (X)	502
5	X (Y) is	208	5	( ) X , Y	412	5	( ) X , Y	272
	X , Y – (	205		X , Y , ,	374		, X , Y ,	242
	X , or Y ,	184		, X , Y ,	334		X , Y , ,	240
	Y , or X ,	166		X (Y) is	291		X (Y) is	209
	Y (X) is	164		, Y , X ,	243		X also known as Y	173
(a)			(b)			(c)		

We observed the number of lexical patterns in the training sets, and the distribution of lexical patterns in different container types is shown in the following tables. Table 5 presents the top 5 lexical patterns for each web page, and Table 6 indicates the top 5 lexical patterns for each website. Table 7 lists the top 5 lexical patterns for each synonym pair. The number of lexical patterns within each container type is different; thus, the ReLPR algorithm performs dissimilarly. That is, the lexical patterns influence the lexical-voting approach.

**Table 5:** Top 5 Lexical patterns for pages.

Page	Pattern Number
http://www.ce.yildiz.edu.tr/mygetfile.php?id=2279	1049
http://alumni.imsa.edu/~keithw/tlex/tlex.data	567
http://samueltoth.com/show/wn2.txt	449
http://cpansearch.perl.org/src/prath/webservice-googlehack-0.04/googlehack/datafiles/nouns_list.txt	297
http://www.the-mathclub.net/site/code/bogglecheat/wordlist	290

**Table 6:** Top 5 Lexical patterns for websites.

Website	Pattern Number
www.glossary.com	2752
www.synonym.com	2638
www.audioenglish.net	2638
thesaurus.infoplease.com	2220
www.lexic.us	1996

**Table 7:** Top 5 Lexical patterns for synonym pairs.

Synonym pair	Pattern Number
**complementary dna_cdna**	5534
**cream_ointment**	5427
**adrenaline_epinephrine**	5359
**autoclave_sterilizer**	5103
**aspirin_acetylsalicylic acid**	4336

**Figure 14:**
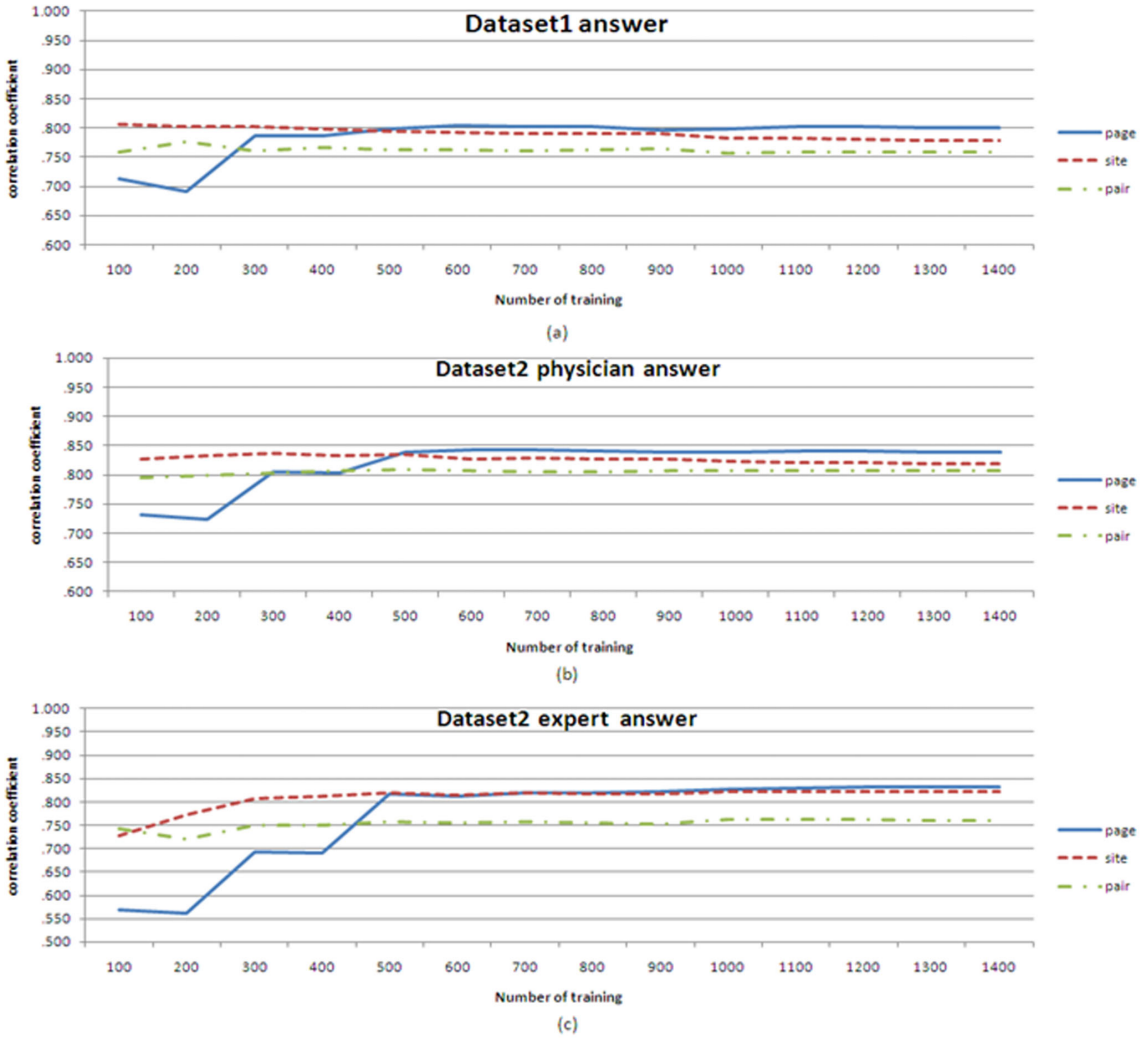
Correlation coefficients of three container types with different training sets.

To test the use of snippets on web pages, we observed how distinct containers varied with the size of the training set. Analyses of correlation coefficients were used to detect the difference among lexical semantic patterns of containers with different sizes. In Figure 14, (a) shows the correlation coefficients for dataset 1 using three approaches. In contrast, (b) and (c) present the correlation coefficients with the manual answers marked by physicians and experts for dataset 2. For the snippets on different containers, the lexical semantic patterns of web pages are significant when the size of training sets is enlarged. In this case, most lexical semantic patterns are generated from the same snippets of a concept pair, and these patterns are similar, such as “Y also known as X”, “Y, known as X”, and “Y, also as X”. Furthermore, using the above experiment, we determined that the optimal size of the training sets was 1400, and the results produced by the training set were used in the following analyses.

### Comparison with other methods

We compared ReLPR with the methods proposed in previous studies. SemDist [27] and Path length [3] are ontology-based methods, while the methods of Leacock & Chodorow [2] and Wu & Palmer [13] are corpus-based approaches. The method of Chen [4] is also a search engine-based approach. Table 8 lists the correlation coefficients of the reported methods for dataset 1. ReLPR outperforms the other methods.

**Table 8:** Correlation coefficients of comparisons using Dataset 1.

Method*	Correlation Coefficient
**SemDist**	0.726
**Path length**	0.422
**Leacock & Chodorow**	0.600
**Wu & Palmer**	0.498
**Chen**	0.798
**ReLPR**	**0.803**

* The first five methods were evaluated in Ref. [4].

Table 9 provides a list of the seven strategies that were used for dataset 2. The results shown in Table 9 indicate that ReLPR performed significantly better than the other seven strategies. In contrast to the previous strategies, the results of ReLPR for both datasets were superior to those of the other strategies when compared with two manual benchmarks.

**Table 9:** Correlation coefficients of comparisons using Dataset 2.

Method*	Physician	Expert
	Correlation Coefficient	Correlation Coefficient
**Path length**	0.512	0.731
**Leacock & Chodorow**	0.358	0.497
**Lin**	0.522	0.565
**Resnik**	0.534	0.610
**Jiang & Conrath**	0.506	0.741
**Vector (All sect, 1Mnotes)**	0.436	0.497
**Chen**	0.705	0.496
**ReLPR**	**0.838**	**0.833**

* The first seven methods were evaluated in Ref. [4].

## Conclusions

In this study, we used the ReLPR algorithm to evaluate the semantic relatedness of two biomedical terms. The ReLPR algorithm estimates semantic relatedness from the lexical patterns of sentence structures and the reinforcing activities between containers and lexical patterns. Our approach is different from previous methods of discovering semantic relations. The approach begins with the automatic identification of lexical patterns and their connections with containers. Then, we construct a synonym lexical pattern database from the snippets of synonym pairs, and we compare the lexical patterns of synonym pairs with those of the queried pairs. Queried pairs that include a higher number of lexical patterns from synonym pairs are more likely to be synonym pairs. Finally, we compared the ReLPR algorithm with previous studies, and the ReLPR algorithm outperformed them.

In future work, we will clarify the influence of lexical patterns on the ReLPR algorithm. More research is needed regarding the relations of different types of lexical patterns. After investigating the lexical patterns of synonym pairs, we believe that the negative relations of concept pairs would improve the performance of the ReLPR algorithm.
